# Patient and Caregiver Perception of Adenoidectomies: A Non‐Real‐World Social Media Analysis

**DOI:** 10.1002/oto2.100

**Published:** 2024-01-25

**Authors:** Nikhil B. Godbole, Ethan D. Paliwoda, Avi A. Gajjar, Nithin Gupta, Alexander Nguyen, Andrew Nguyen, Richard Alexander

**Affiliations:** ^1^ Department of Otolaryngology Tulane University School of Medicine New Orleans Louisiana USA; ^2^ Department of Otolaryngology Albany Medical College Albany New York USA; ^3^ Department of Neurosurgery Hospital of University of Pennsylvania Philadelphia Pennsylvania USA; ^4^ Department of Otolaryngology Campbell University School of Osteopathic Medicine Lillington North Carolina USA; ^5^ Department of Otolaryngology Creighton University School of Medicine Phoenix Arizona USA; ^6^ Department of Otolaryngology, College of Medicine University of Florida Gainesville Florida USA; ^7^ Granville ENT Oxford North Carolina USA

**Keywords:** adenoid, adenoidectomy, caregiver, Instagram, patient perception, social media

## Abstract

**Objective:**

To survey the social media outlets Twitter and Instagram for public posts related to adenoidectomy surgery. This study aims to investigate the attitudes and perceptions of patients and caregivers on social media, through thematic content‐analysis of social media posts regarding adenoidectomy.

**Study Design:**

Non‐real world qualitative study.

**Setting:**

Twitter and Instagram social media platforms.

**Methods:**

Public posts uploaded between February, 2021 and February, 2023 using the hashtags “#adenoidectomy,” and “#adenoidectomyrecovery” were searched. Posts were excluded if they were unrelated to adenoidectomy or were in a non‐English language. Relevant posts were stratified demographically as patient or caregiver and pre‐ or postoperative, and categorized into relevant themes for analysis. Outcomes were measured as the total number of posts.

**Results:**

A total of 394 relevant posts were analyzed. A significance threshold of *P* < 0.05 was used. Patients posted significantly more posts regarding procedure pain (*P* = 0.002) and concern for appearance (*P* = 0.048) compared to caregivers. Caregivers posted significantly (*P* < 0.001) more posts regarding condition awareness and were significantly (*P* < 0.001) more likely to spread positivity in their posts compared to patients themselves. Posts made by female caregivers were more likely to reference fear, while those made by male caregivers were more likely to provide education (*P* = 0.002).

**Conclusion:**

Patients may worry about appearance and mental health while caregivers are more likely to spread information and positivity. Male and female caregivers may also use social media differently. A better understanding of patient and caregiver concerns may optimize physician interaction and involvement.

Social media is defined as internet‐based platforms that allow for the engagement and exchange of user‐generated content.^1^ With 72% of all adult Americans currently using social media and more Americans gravitating toward it, social media's user base is more representative of the broader population than ever before.^1^ Outlets such as Twitter and Instagram are instantly accessible sources of beliefs, opinions, and information.

Surgical interventions in the pediatric population can be a source of anxiety for both children and their parents. For children, fear of the unknown and separation from their parents can contribute to their worry.^2^ Parents may feel helpless, concerned about the procedure and possible complications, and stressed about managing postoperative care.^3^ As such, it is important to consider the mental health of both the child and their parent when undergoing any surgical interventions.

Patient feedback trends among patients and caregivers in pediatric populations has not been thoroughly investigated. Social media allows for the exchange of patient information through a provider‐independent medium, eliminating numerous limitations of surveys administered in clinical settings, such as time‐lag and favorability bias.^4^ This unique platform also circumvents many of the limitations posed by traditional real‐world qualitative research design, including researcher bias and response bias. Studies have shown that patients utilize social media due to their health care provider's inability to fulfill certain emotional and informational needs.^4^ Additionally, recent studies have demonstrated social media usage among a variety of medical and surgical patient populations. However, none have explored its usage in relation to common otolaryngologic procedures such as adenoidectomy.^5^, ^6^

Adenoidectomy is one of the most commonly performed surgeries in children, with over 500,000 procedures performed annually in those <15 years of age.^7^ Although the indications and procedure itself are extremely common, it has been shown that online information about the procedure is inadequate for properly educating patients.^8^ Further, there is a relative paucity of literature regarding the perceptions of both caregivers and patients on adenoidectomies.

Social media analysis allows health care providers to gain a holistic understanding of patient experience as well as a more direct insight into positive and negative attitudes regarding a procedure.^9^ As adenoidectomies are extremely common, this study seeks to understand the affective state of patients and caregivers outside of the health care setting by incorporating data outside the scope of an in‐clinic encounter. In doing so, this investigation will provide crucial insight that can supplement and enhance shared decision‐making among patients, parents, and the health care team improving counseling for pediatric adenoidectomy.

## Methods

A non‐real‐world social media content analysis was performed. Social media outlets Twitter and Instagram were searched to find posts relating to the patient and caregiver experience of adenoidectomy surgery. Posts were demographically stratified by gender, identity of poster, and themes. Since this study utilized publicly obtained social media posts, it was exempt from review per the standing policy of the Campbell University Institutional Review Board.

Twitter and Instagram were queried with the hashtags “#adenoidectomy” and “#adenoidectomyrecovery” to reveal posts pertaining to adenoidectomy surgery. Instagram allows for captioned photos and videos while Twitter allows for primarily text media with occasional photos and videos. Posts were included whether they were made by a caregiver or the patient themselves. Non‐patient‐specific posts were excluded. This study follows COnsolidated criteria for REporting Qualitative research guidelines (Supplemental Table S1, available online).^10^

Social media posts uploaded between the dates of February 2021 to February 2023 were searched. The Twitter search revealed no related posts. The Instagram search revealed 12,000 posts. Of these 11,606 posts were eliminated by 3 investigators (NBG, EDP, AAG). Posts were excluded if they did not specifically pertain to an individual's (patient or caregiver) experience of adenoidectomy. Posts made within the pre‐ or postoperative period were eligible for inclusion. This elimination process yielded 394 eligible posts.

The 394 posts were reviewed by 2 investigators (N.B.G., A.A.G.). A cursory review of the included posts was performed without any coding to develop a list of preliminary themes. The preliminary themes were deemed suitable and no additional themes were added. The frequency of major themes was assessed as the number of posts (N). The profile of the poster was then searched to determine if the post was made preoperatively or postoperatively. Postoperative posts which included a description of the preoperative experience were classified as both. The outcome measure of interest was the total number of posts (N). Due to the nature of social media, including limited available demographic data, a quantifiable number of individual subjects with clearly identified demographic characteristics was not obtainable.

Data saturation was considered achieved when all reported posts under the aforementioned hashtags were either included in one of the listed categories or excluded according to previously defined exclusion criteria.^11^

The frequency of each theme was assessed as the total number of posts (N). Categorical variables were shown as proportions and compared using either *χ* ^2^ tests or Fisher's exact test depending on the normality of the data. Statistical analyses were conducted with STATA V17.0 software with a *P*‐value set at 0.05 for significance. A *P* < 0.05 indicated a theme was of relative importance for patients when compared to caregivers or vice versa.

## Results

### Qualitative Analysis/Study Set

Initial screening of adenoidectomy surgery‐related posts affiliated specifically with either individual patients or caregivers yielded 394 posts appropriate for analysis. The 394 posts were further categorized based on demographics including gender of the poster, gender of the posting caregiver, whether the poster was the patient or the caregiver, and whether the post pertained to preoperative or postoperative concerns. Additionally, posts were categorized by theme based on the criteria outlined in Table 1.

**Table 1:** Coding Tree of Criteria Defining Themes Pertaining to Adenoidectomy Social Media Posts

Theme	Criteria
Appearance of nasal cannula	Image/text specifically referencing presence of nasal cannula
Appearance of G tube	Image/text specifically referencing presence of G tube
Recovery/rehabilitation	Image/text specifically discussing the recovery/rehabilitation process postoperatively
Sleeping issues	Image/text specifically discussing issues of sleep onset or maintenance in the context of the procedure
Recounts other symptoms/complications	Image/text specifically discussing associated symptoms or surgical complications in the context of the procedure
Eating/drinking issues	Image/text specifically referencing issues ingesting solids or liquids in the context of the procedure
Pain	Image/text specifically referencing experience of pain preop or postop
Most difficult part of experience	Image/text with specific reference to the most challenging aspect in the context of the procedure
How experience made them stronger or more resilient	Image/text specifically discussing improvements in character growth, strength, or resilience in the pre or postoperative period
Awareness of condition	Image/text intended to disseminate information regarding risk factors, onset, or treatment of the condition to educate the general public
Spreading positivity	Image/text sharing positive or encouraging messages in the context of the procedure
Religious connotations	Image/text containing specific mentions of religion or God before or after the procedure
Life satisfaction/quality of life	Image/text specifically containing positive depictions or descriptions of changes to quality of life or ability to enjoy life in the postoperative period
Mental health of patient	Image/text describing or depicting the mental health of the patient specifically
Mental health of social media poster	Image/text specifically describing or depicting the mental health status of the social media poster regardless of identity
Scientific explanation of condition	Post containing a clinical or scientific explanation of the condition using text or visual images
Online support	Image/text containing explicit mentions of seeking or utilizing online support
Concern of appearance	Image focusing on physical appearance or written/verbal text on current physical appearance prior to surgery
Satisfaction with healing process	Image/text specifically discussing progression of postoperative healing using positive descriptors in text
Fear of procedure	Image/text demonstrating concern, fear, worry, or stress in the preoperative context of the procedure
Update of appearance while recovering from treatment	Image focusing on physical appearance or written/verbal text on changes to physical appearance as a result of surgery postoperative
Covid 19	Image/text specifically discussing COVID‐19 pandemic in the context of the procedure

### Synthesis of Results

Counts and proportions (as percentages) of posts satisfying each aforementioned category were calculated and further stratified in Tables 2 and 3 by whether the poster was the patient or the caregiver and whether the gender of the posting caregiver was male or female.

**Table 2:** Demographic Characteristics and Themes of Adenoidectomy Social Media Posts^a^

	Patient themselves N (%)	Caregiver themselves N (%)	*P* value	Male caregiver posting	Female caregiver posting	*P* value
**Gender**			**Male vs female**			
Male	8 (17.8%)	195 (55.1%)	<0.001**	46 (13.0%)	**‐**	**‐**
Female	37 (82.2%)	154 (43.5%)	<0.001**	**‐**	276 (78.0%)	**‐**
**Surgery time**			**Preoperative vs postoperative**			**Preoperative vs postoperative by caregiver gender**
Preop	6 (13.3%)	100 (28.3%)	0.033*	15 (15.6%)	72 (26.1%)	0.711
Postop	39 (86.7%)	254 (71.8%)	0.033*	30 (65.2%)	200 (72.5%)	0.711

Abbreviation: N, number of posts.

^a^ *Denotes significance at at *P* = 0.05, **denotes significance at *P* = 0.01.

* Analysis of gender of patient and themes was not done due to low sample size likely skewing statistical results.

** Gender was unknown for some individuals hence total percentage may not equal 100%.

**Table 3:** Themes of Adenoidectomy Social Media Posts^a^

Theme	Patients themselves N (%)	Caregivers themselves N (%)	Patients vs caregiver (*P* value)	Male caregivers posting	Female caregivers posting	Male vs female caregivers (*P* value)
Appearance of nasal cannula	4 (8.9%)	14 (4.0%)	0.619	1 (2.2%)	12 (4.4%)	0.488
Appearance of G tube	1 (2.2%)	10 (2.8%)	0.14	0	0	0.214
Recovery/rehabilitation	17 (37.8%)	157 (44.4%)	0.618	19 (41.3%)	127 (46.0%)	0.552
Sleeping issues	9 (20.0%)	99 (28.0%)	0.562	11 (23.9%)	81 (29.4%)	0.450
Recounts other symptoms/complications	10 (22.2%)	87 (24.6%)	0.427	9 (19.6%)	69 (25.05%)	0.426
Eating/drinking issues	14 (31.1%)	53 (15.0%)	0.897	6 (13.0%)	44 (15.9%)	0.615
Pain	14 (31.1%)	51 (14.4%)	0.002**	6 (13.0%)	45 (16.3%)	0.575
Most difficult part of experience	6 (13.3%)	44 (12.4%)	0.102	3 (6.5%)	41 (14.9%)	0.128
How experience made them stronger or more resilient	5 (11.1%)	24 (6.8%)	0.158	1 (2.2%)	23 (8.3%)	0.141
Awareness of condition	5 (11.1%)	67 (19.0%)	<0.001**	11 (23.9%)	52 (18.8%)	0.422
Spreading positivity	16 (13.6%)	131 (37.0%)	<0.001**	24 (52.2%)	94 (34.1%)	0.018**
Religious connotations	5 (11.1%)	52 (14.7%)	0.795	3 (6.5%)	43 (15.6%)	0.104
Life satisfaction/quality of life	11 (24.4%)	82 (23.2%)	0.004**	10 (21.7%)	67 (24.3%)	0.709
Mental health of patient	14 (31.1%)	31 (8.8%)	<0.001**	4 (8.7%)	24 (8.7%)	1.000
Mental health of social media poster	15 (34.9%)	62 (18.3%)	0.037*	1 (2.3%)	59 (22.1%)	0.002**
Scientific explanation of condition	0	16 (4.5%)	0.036*	6 (13.0%)	8 (2.9%)	0.002**
Online support	0	23 (6.5%)	0.071	3 (6.5%)	18 (6.5%)	1.000
Concern of appearance	2 (4.4%)	4 (1.1%)	0.048*	0	4 (1.5%)	0.411
Satisfaction with healing process	10 (22.2%)	103 (29.1%)	0.001**	14 (30.4%)	81 (29.4%)	0.881
Fear of procedure	4 (8.9%)	60 (17.0%)	0.018*	0	56 (20.3%)	0.001**
Update of appearance while recovering from treatment	14 (31.1%)	124 (35.0%)	0.689	9 (19.6%)	103 (37.3%)	0.019
Covid 19	4 (8.9%)	21 (5.9%)	0.743	1 (2.2%)	18 (6.5%)	0.247

Abbreviation: N, number of posts.

^a^ *Denotes significance at *P* = 0.05, **denotes significance at *P* = 0.01.

* Analysis of gender of patient and themes was not done due to low sample size likely skewing statistical results.

** Gender was unknown for some individuals hence total percentage may not equal 100%.

### Demographics (Table 2)

In total, 394 posts were analyzed in the data set. The majority of individuals posting regarding adenoidectomy surgery were caregivers (349; 88.6%). Additionally, most posts were made by females (276; 78.0%). The majority of posts were made postoperatively (293; 73.4%). Caregivers were more likely to post preoperatively whereas patients were more likely to post postoperatively (*P* < 0.033).

### Themes (Table 3)

**Figure 1:**
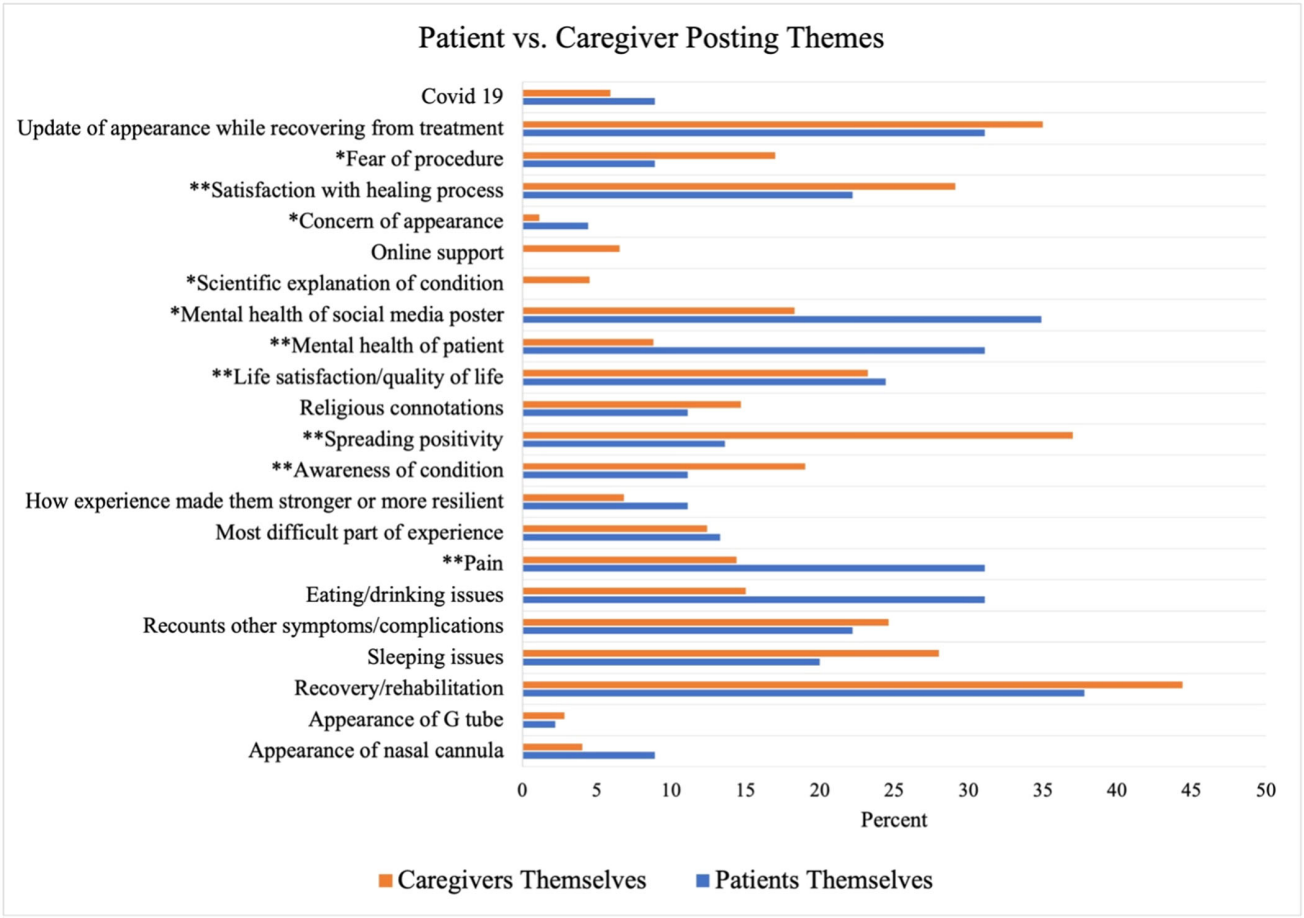
Graphical representation and statistical comparison of patient versus caregiver posting themes. *P = 0.05 and **P = 0.01.

The most common theme among posts made by patients themselves was recovery/rehabilitation (37.8%). On the other hand, the least common themes among these posters were scientific explanation of conditions (0%) and reaching out/offering online support (0%). The most common theme among caregiver posts was recovery/rehabilitation (44.4%). The least common theme among these posters was concern of appearance (1.1%) (Figure 1).

### Statistical Analysis

Probable statistical relationships were shown to exist between the following categories as seen in the *P*‐value columns of Tables 2 and 3. Patients posted significantly more posts regarding procedure pain compared to caregivers (*P* = 0.002). Caregivers posted significantly (*P* < 0.001) more posts regarding condition awareness and were significantly (*P* < 0.001) more likely to spread positivity in their posts compared to patients themselves. The effect of gender on spreading positivity demonstrated that male caregivers were significantly (*P* < 0.018) more likely to make such posts compared to female caregivers. Patients themselves (24.4%) were significantly more likely to post about life satisfaction or quality of life (*P* = 0.004), about the mental health of patients (*P* < 0.001), and about the mental health of the social media poster (*P* = 0.037) compared to caregivers.

**Figure 2:**
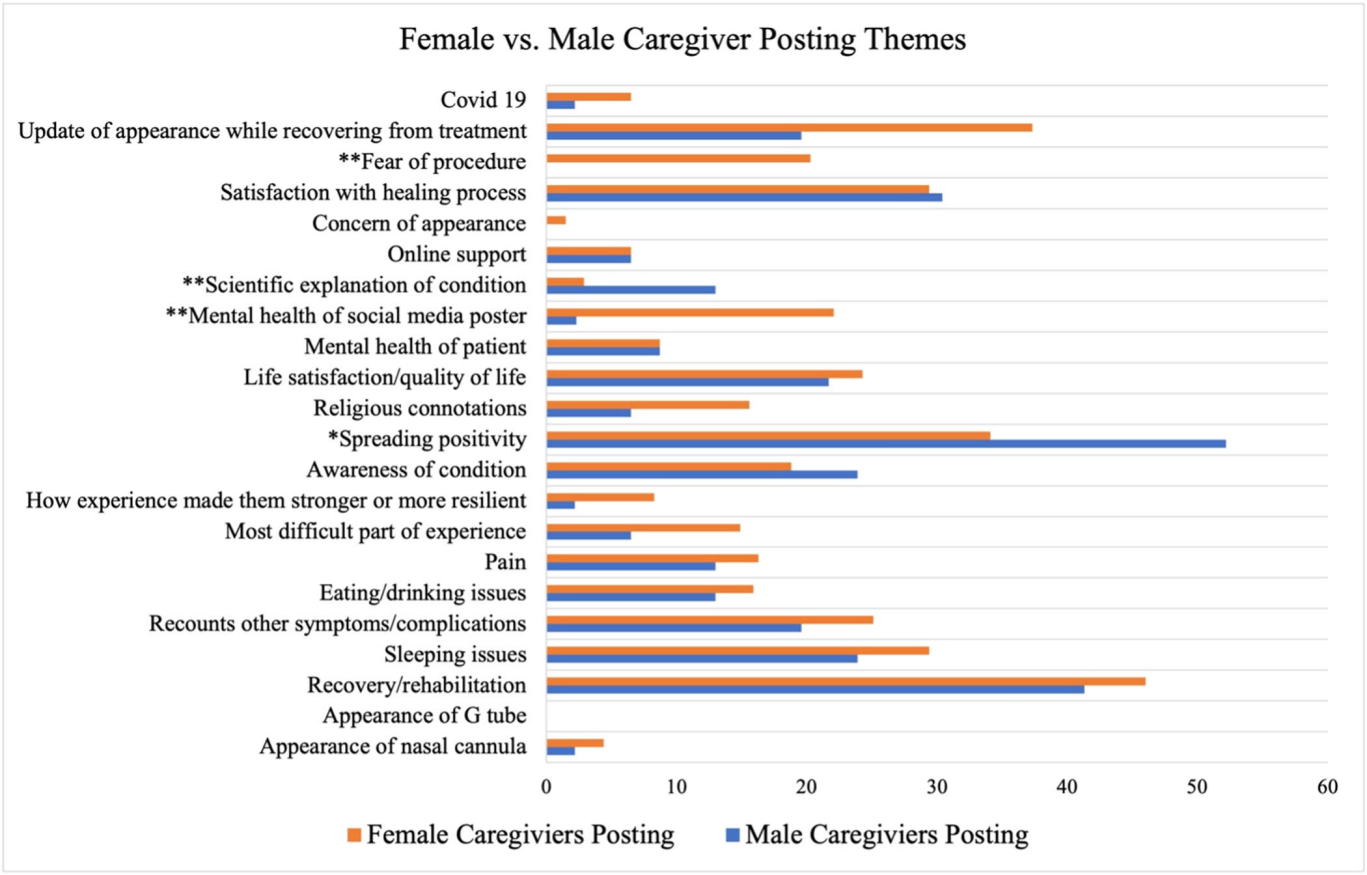
Graphical representation and statistical comparison of female versus male caregiver posting themes. *P = 0.05 and **P = 0.01.

Sex differences among caregivers were also analyzed. The effect of gender on the fear of the procedure was evaluated, showing that female caregivers (20.3%) were significantly (*P* = 0.001) more likely to make such posts compared to male caregivers (0%). Female caregivers (22.1%) were significantly (*P* = 0.002) more likely to make posts related to mental health compared to male caregivers (2.3%). Caregivers (4.5%) were significantly (*P* = 0.036) more likely to post scientific explanations of the condition compared to patients. Furthermore, male caregivers (13%) were significantly (*P* = 0.002) more likely to make scientific explanation posts compared to female caregivers (2.9%) (Figure 2).

Patients themselves were significantly (*P* = 0.048) more likely to post with concerns of physical appearance. Caregivers were significantly more likely to post about their satisfaction with the healing process (*P* = 0.001) and were significantly more likely to post regarding fear of the procedure (*P* = 0.018) than patients themselves. Due to the low quantity of posts made by patients, sex differences analysis was not conducted on this population.

## Discussion

The literature indicates that dissonance between the priorities of physicians and those of patients and their families remains a significant source of dissatisfaction among patients.^9^, ^12^ Thus, this study sought to examine perceptions through the lens of social media. In doing so, this study provides a unique perspective on aspects of patient care that may be improved.

### Gender Differences

Gender was found to influence the focus of social media posts with female caregivers more likely to post about the mental health of the social media poster and fear of the procedure and male caregivers more likely to post about the scientific explanation of the condition and spreading positivity. By focusing on mental health and emotions of fear regarding adenoidectomies, female caregivers may be empathizing with their family members to help mitigate the stresses of surgery. Woitowitch et al identified a similar gender relationship in social media use among providers.^13^ The findings of this study suggest that male caregivers may be more likely to post with the intention of spreading their own knowledge on the medical condition. Female caregivers, on the contrary, were found to utilize social media with a stronger intention of building a support network in the online caregiver community. Similar trends have been observed in other studies assessing the use of social media by female caregivers.^14^ Conversely, male caregivers may utilize social media to provide clinical education and promote condition awareness. A similar social media analysis of patients with cerebral aneurysms identified similar trends in which male patient focus was placed on raising awareness of their condition.^15^

### Patient and Caregiver Perceptions

Concerns voiced on social media were found to differ depending on the identity of the poster. Patients were more likely to post postoperatively. Patient posts were more likely to contain themes of mental health, eating/drinking issues, pain, and appearance while recovering from treatment. Caregivers were more likely to post preoperatively. Caregiver posts were more likely to contain themes of spreading positivity regarding the condition, update of appearance while recovering from treatment, satisfaction with healing process, and sleeping issues. Both patient and caregiver posts contained themes of functional recovery/rehabilitation.

**Figure 3:**
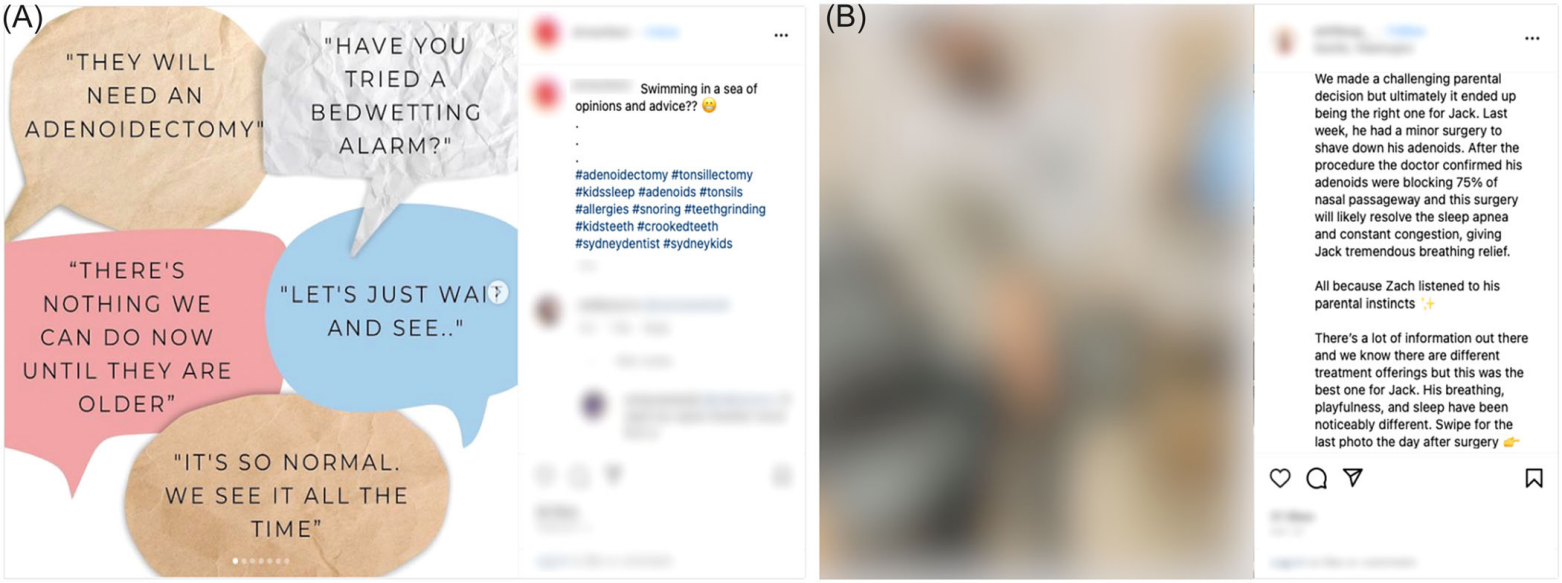
Example Instagram posts (A, B) depicting caregiver anxiety related to adenoidectomy.

As a whole, patients were more likely to voice concerns about appearance and mental health while caregivers were more likely to express satisfaction with the healing process and spreading positivity. Although caregivers tended to be satisfied with good outcomes, several of the posts generated from our search depicted anxiety associated with the medical decision‐making preadenoidectomy similar to the posts included in Figure 3. The first post in Figure 3 highlights the mental health concerns of parents of adenoidectomy patients encompassing the surgery. Specifically, the post mentions the parental anxiety and instinct associated with pursuing second opinions for children. Other posts highlight realistic discouragement in inquiring about or pursuing adenoidectomy. The second post emphasizes the importance of asking questions as a means to alleviate anxiety when it comes to concerned parents. Negative feelings such as depression and anxiety surrounding adenoidectomy are probable when faced with complications, as observed by Zagolski and Kulisiewicz.^16^ This information highlights the lack of interventions in place to console parents of patients undergoing procedures such as adenoidectomy.

Social media posts from pediatric adenoidectomy patients are more limited compared to caregiver posts, however, these effects have been detailed in the literature. Tunney and Boore demonstrated significant alleviation of preoperative anxiety in children undergoing tonsillectomy and adenoidectomy before and after being given a standardized storybook compared to control children who received a coloring book.^17^ Similarly, the primary emotion in preschool age children was determined to be fear in a study by Fukuchi et al.^18^ Efficacy in utilizing a coping method such as a storybook suggests an opportunity to curtail the negative associations for patients undergoing these procedures.

These findings reveal a trend of negative perception surrounding adenoidectomy among patients on social media. Themes of pain, scarring, and mental health were significant associations.

Similar social media analyses have identified common themes concerning pain and appearance to be a notable source of concern surrounding a procedure.^19^ These results also suggest that scarring may be a significant quality‐of‐life concern among adenoidectomy patients.

Interestingly, there may be a relationship between the prevalence of negative themes and the type of procedure. This suggests that a patient‐centered understanding of the unique challenges of a procedure or condition may be central to effective patient care. In the literature, anterior cervical discectomy and fusion surgery were found to have a predominantly positive perception, while surgery to correct male infertility had a predominantly negative perception.^20^, ^21^

### Relevance for Health Care Providers

To provide effective patient‐centered care, in‐clinic counseling should appropriately prioritize patient and caregiver concerns. Health care providers may consider counseling patients and caregivers to engage with social media platforms to identify individuals with shared experiences. Exposure to additional patient perspectives of the postoperative period through social media may also provide critical insight into the process of functional recovery and rehabilitation. Ultimately, this could guide in‐clinic counseling with time‐appropriate points of focus for counseling, within each stage of the operative period.

Literature examining social media in various surgical procedures advocates incorporating educational content into provider social media posts.^22^, ^23^ This could assuage such issues surrounding patient education through digestible and accessible material sourced directly from the health care team. Some authors also suggest utilizing social media to “demystify” the operating room and postoperative anesthesia units by depicting information about surgical equipment, patient testimonials, and intraoperative photos and videos.^9^ Providers may consider volunteering resources to online social support groups and educational tools and resources promoting awareness to male and female caregivers alike.

### Limitations

Social media‐based studies are prone to intrinsic limitations. These challenges include determining the precise timing of posts during the pre‐ or postoperative period, variations in procedure types, and limited demographic data. Additionally, nonstandard measures of patient data and potential human errors add further challenges to obtaining generalizable findings. To mitigate this, a written data collection guideline was prepared for this project and used by all authors.

Social media posts are ultimately a convenience sample and are thus limited in interpretation to the domains of Instagram and Twitter. Due to privacy settings, the full subset of adenoidectomy‐related social media posts may not have been accessible. Additionally, not all patients who underwent adenoidectomy post about it. This may be especially true as many of the patients undergoing adenoidectomies are children. Furthermore, patients, let alone pediatric patients, often lack complete comprehension of their condition and thus would benefit from targeted educational interventions to increase knowledge as well as promote shared decision‐making.^24^ Thus, distinguishing between patients who received adenotonsillectomies and those who received only adenoidectomies remains a limitation of this social media analysis.

Adenotonsillectomy is one of the most common pediatric‐performed procedures.^7^ This sample represents only a small fraction of the total annual number of adenoidectomies performed, emphasizing the need for a greater representation of the total number of patients undergoing this procedure annually. Additionally, there is a demographic bias as caregivers and patients who engage in online communities are not representative of the entire set.

Similar studies suggest that social media analysis may select for experiences involving strong emotions, such as fear and pain.^19^, ^25^ Social media analysis is also limited by the lack of clinical verification of the posts. Additionally, posts may be made by bots or electronic groups. Similarly, as recorded data is retrospective and anonymous, posts cannot be returned to posters for clarification. As a result, details regarding long‐term consequences not explicitly stated in the post are not obtainable. This report also is confined by the limitations of qualitative research.

The unique advantages of social media analysis add a unique perspective to existing literature. Most notably, response bias is minimized. Because this is a non real‐world study, there is no relationship between subjects and researchers. There is also less systematic error due to variation in researcher training or phrasing of leading questions. Social media provides a unique platform for gathering patient perspectives throughout the course of treatment, as patients and caregivers frequently post their candid thoughts regarding the procedure.

### Future Directions

Further investigation into patient, caregiver, and gender‐specific perceptions of the surgical experience on social media is warranted. Primary data from interviews and focus groups should be collected to better understand long‐term emotional points of concern, compare preoperative concerns with postoperative concerns, and derive procedure‐specific provider recommendations. Finally, follow‐up studies should also evaluate the therapeutic effects of social media participation on feelings of tension and anxiety in patients and caregivers following surgery.^26^

## Conclusion

This study finds that as a whole, patients were more likely to voice concerns about appearance and mental health while caregivers were more likely to express satisfaction with the healing process and to spread positivity. Furthermore, the study findings suggest that female patients and caregivers may trend toward utilizing social media for social support whereas male patients and caregivers may have a tendency to seek increased awareness of the medical condition and procedural education. These findings should be used to inform conversation during the perioperative period to further homogenize the patient‐provider relationship. Finally, physicians should consider patient and caregiver concerns to inform patient‐centered counseling, thus leading to increased patient satisfaction.

## Author Contributions

**Nikhil B. Godbole**, study design, writing of original draft, and editing of final drafts; **Ethan D. Paliwoda**, writing of original draft, editing of final drafts; **Avi A. Gajjar**, project conceptualization, statistical analysis, data collection, writing of original draft and editing of final drafts; **Nithin Gupta**, editing of final drafts; **Alexander Nguyen**, editing of final drafts. **Andrew Nguyen**, editing of final drafts; **Richard Alexander**, reviewing, editing, and supervision.

## Disclosures

### Competing interests

None.

### Funding source

This research did not receive any specific grant from funding agencies in the public, commercial, or not‐for‐profit sectors.

## Supporting information


**Supplemental Table 1**. Consolidated criteria for Reporting Qualitative research (COREQ) checklist with identified page numbers.
